# Coronary Artery Bypass Graft During the COVID-19 Pandemic

**DOI:** 10.21470/1678-9741-2020-0283

**Published:** 2020

**Authors:** Lucas Molinari Veloso da Silveira, Gustavo Pampolha Guerreiro, Luiz Augusto Ferreira Lisboa, Omar Asdrúbal Vilca Mejía, Luís Roberto Palma Dallan, Luís Alberto Oliveira Dallan, Fabio B Jatene

**Affiliations:** 1 Cardiovascular Surgery Division, Instituto do Coração do Hospital das Clínicas da Faculdade de Medicina da Universidade de São Paulo (InCor-HCFMUSP), São Paulo, SP, Brazil.

**Keywords:** Cardiac Surgical Procedures, COVID-19, Coronavírus, Pandemia, SARS Virus, Perioperative Period

## Abstract

Since the beginning of the coronavirus disease (COVID-19) pandemic, in March 2020, the number of people infected with COVID-19 worldwide increases continuously. Brazil is being followed with great concern in the international media, as it can, very soon, be the epicenter of the pandemic. Initial surgical data suggest that patients who acquire COVID-19 in the perioperative period are prone to a higher morbidity and mortality, however, evidence in cardiac surgery is still scarce.

This article aims to aggregate to the growing evidence suggesting that perioperative infection with severe acute respiratory syndrome coronavirus 2 contributes to a more morbid evolution of the case.

**Table:** 

Abbreviations, acronyms & symbols	
COVID-19	= Coronavirus disease
CPB	= Cardiopulmonary bypass
ICU	= Intensive care unit
POD	= Postoperative day
SARS-CoV-2	= Severe acute respiratory syndrome coronavirus 2
STS	= Society of Thoracic Surgeons

## INTRODUCTION

Since December 2019, the world is facing a unique situation: the coronavirus disease (COVID-19). COVID-19 is a viral infection caused by severe acute respiratory syndrome coronavirus 2 (SARS-CoV-2), considered a pandemic since March 11^th^, 2020. As of May 19^th^, 2020, there have been 4,731,458 confirmed cases and 316,169 deaths worldwide^[1]^. In Brazil, the first case was confirmed in February 26^th^, 2020. Currently, Brazil is being considered the future epicenter of the pandemic, with 241,080 confirmed cases and 16,118 deaths until May 19^th^, 2020^[1]^.

As physicians are passing through real-time learning process on the management of COVID-19, patients continue to present with other medical conditions. Unstable cardiovascular disease is one of these conditions, sometimes requiring urgent treatment.

Physicians will have to balance the risk of delaying medical (especially surgical) treatment for patients with significant cardiovascular disease-associated with high mortality in COVID-19 patients^[2]^-against the risk of patients being infected with SARS-CoV-2 during the perioperative period and its consequences^[3]^.

There are few case reports of patients who underwent cardiac surgery and were diagnosed with COVID-19. The aim of this article is to draw the attention of adult cardiac surgeons and cardiologists regarding the management of patients considered for surgical procedures in the context of the current COVID-19 pandemic.

## SCENARIO

A 63-year-old male patient with hypertension was admitted to the emergency room with non-ST-segment elevation myocardial infarction. Coronary angiogram showed severe multivessel coronary disease with a high syntax score (35.5). Transthoracic echocardiography showed preserved left ventricle ejection fraction and no other abnormalities. The calculated values of risk scores were: EuroSCORE II 1.62% and STS score 0.97%. Based on the recommendations of the Brazilian health government organizations at the time, preoperative screening for COVID-19 was not performed.

Surgical procedure was performed 4 days after the patient’s admission. The patient underwent CABG via full sternotomy, with cardiopulmonary bypass (CPB) and myocardial protection with intermittent cold blood cardioplegia. The targeted grafts were: left internal mammary artery to the left anterior descending artery and saphenous veins grafts to two diagonal branches and an obtuse marginal branch. The right coronary territory was not grafted due to poor targets. The patient was weaned from CPB without difficulty. After completion of the procedure, the patient was transferred to the intensive care unit (ICU)-timeline in Figure 1.

**Fıg. 1:**
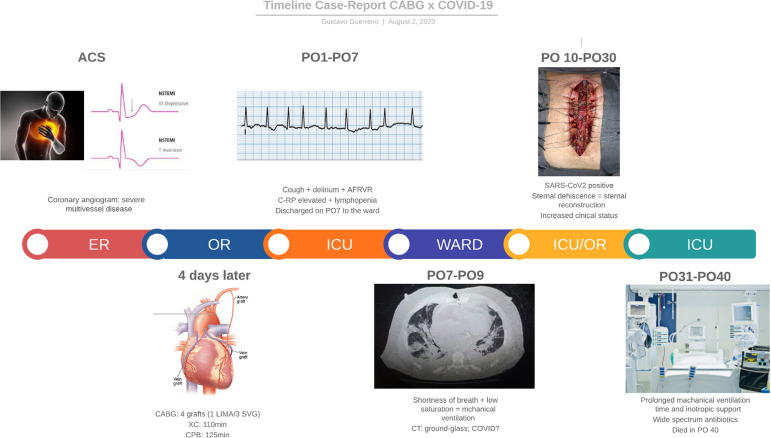
Timeline of events.

**Fig. 2:**
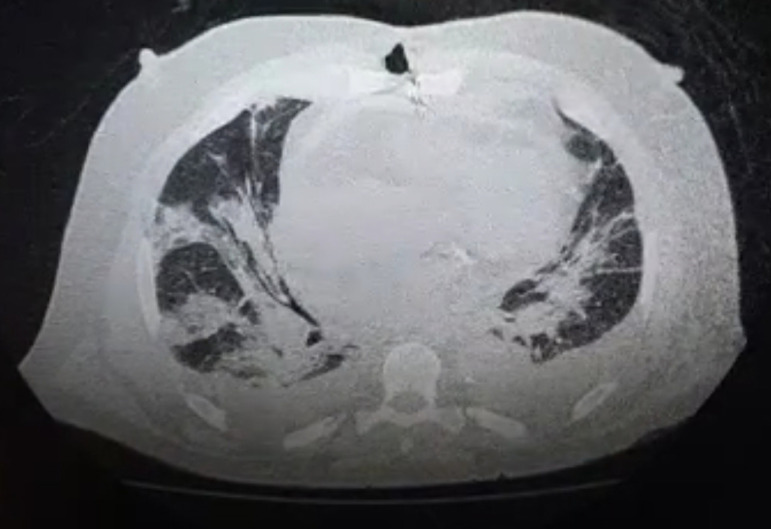
Chest CT revealing multiple peripheral ground-glass opacities.

The initial postoperative course in ICU was uneventful. On postoperative day (POD) 4, the patient developed cough and atrial fibrillation with rapid ventricular response, which was controlled with amiodarone. Laboratory tests disclosed an increase in C-reactive protein of 147.50 mg/L and 5730 leukocytes/mm^[3]^ with lymphopenia-516 lymphocytes/mm^[3]^. On POD 9, he had shortness of breath and oxygen saturation of 78%. A chest computed tomography scan revealed multiple peripheral ground-glass opacities in both lungs (Figure 2 and Video 1). Quantitative reverse transcription-polymerase chain reaction for coronavirus was positive. The patient was transferred to the COVID-ICU and required mechanical ventilation.

**Video 1:**
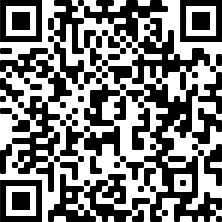
Chest CT revealing multiple peripheral ground-glass opacities.

In the COVID-ICU, he had difficulty breathing and multiple complications. The sternum became unstable and a small dehiscence of the lower segment of the sternal wound was evident, but without local signs of infection. Debridement of sternal and subcutaneous tissues plus sternal refixation and installation of negative pressure wound therapy system were performed. Concerning his sternal wound, the patient showed a remarkable evolution and, 6 days after the first procedure, he underwent reconstruction of the sternal wound with a pectoralis muscle flap. The patient presented a favorable evolution with weaning from inotropic drugs and extubation. However, a new worsening, requiring a new intubation, occurred due to secondary pulmonary infection.

The patient persisted in the ICU with prolonged use of inotropes and mechanical ventilation, as well as multiple clinical complications until POD 40, when he died due to pulmonary infection and multiple organ failure.

## DISCUSSION

Cardiac surgery has been particularly affected by the coronavirus outbreak since elective surgeries were cancelled and ward and ICU beds dedicated to cardiac surgery were reassigned to COVID-19 patients^[4]^. Surgeons and cardiologists are facing a series of difficult decisions to be made, since it is necessary to balance the risk between postponing surgery in patients with significant cardiovascular disease and the risk of operating a patient in latent period of COVID-19 or acquiring nosocomial COVID-19 infection.

Data on surgical outcomes of patients with COVID-19 are rare. Lei et al.^[5]^ reported data from 34 patients who underwent different surgeries during the incubation period of COVID-19 and suggested that surgery may exacerbate disease progression, since the mortality rate in his cohort-20.6%-is higher than in surgical patients without COVID-19 as well as in COVID-19 patients without surgery. This may be related to impaired immune function^[6]^ and early systemic inflammatory response due to surgery^[7]^.

Patients who need cardiac surgery are likely to have a more severe presentation of COVID-19, due to their underlying cardiovascular disease, which is associated with high mortality in COVID-19^[2]^. It is also suggested that these patients may be more susceptible to myocardial disfunction-a possible complication of COVID-19-after surgery, especially if cardioplegic arrest is used^[8]^.

Currently, data on COVID-19 and cardiac surgery results are almost inexistent; there is only one report of in-hospital infection of a patient submitted to a CABG who died in POD 9 with COVID-19 pneumonia as a presumed cause of death^[9]^. Despite the same outcome occurred in both cases, the death of our patient was due to indirect consequences-nosocomial infections-from prolonged hospital length of stay in COVID-19.

Due to the current situation, many scientific societies have published guidance statements for cardiac surgery institutions. Recommendations are based on inpatient COVID-19 load and operative capacity^[3]^. Aiming to reduce perioperative morbidity, all patients should be tested preoperatively, and a positive result should elicit a Heart Team evaluation about the urgency of the case. Patients should be operated as soon as possible to reduce possible SARS-CoV-2 nosocomial infection. In this situation, some patients could be operated before the end of the period of discontinuation of antiplatelet medications.

This article adds evidence of a poor outcome in a lowrisk urgent CABG patient infected with SARS-CoV-2 in the perioperative period. Unfortunately, the patient was not tested preoperatively, and hence we cannot affirm that he was already infected when operated on. During COVID-19 pandemic, patients are liable to a more morbid postoperative course, reinforcing the importance of Heart Team evaluation for choosing the best treatment option, even in low-risk patients.

**Table:** 

**Authors' roles & responsibilities**	
LMVS	Substantial contributions to the conception or design of the work; or the acquisition, analysis, or interpretation of data for the work; final approval of the version to be published
GPG	Substantial contributions to the conception or design of the work; or the acquisition, analysis, or interpretation of data for the work; final approval of the version to be published
LAFL	Drafting the work or revising it critically for important intellectual content; final approval of the version to be published
OAVM	Final approval of the version to be published
LRPD	Final approval of the version to be published
LAOD	Final approval of the version to be published
FBJ	Final approval of the version to be published
